# Healthcare and sociodemographic conditions related to severe maternal morbidity in a state representative population, Federal District, Brazil: A cross-sectional study

**DOI:** 10.1371/journal.pone.0180849

**Published:** 2017-08-03

**Authors:** Douglas dos Santos Moreira, Muriel Bauermann Gubert

**Affiliations:** Department of Nutrition, Faculty of Health Sciences, University of Brasília, Brasília, Distrito Federal, Brazil; Universiti Sains Malaysia, MALAYSIA

## Abstract

**Background:**

The concept of severe maternal morbidity (SMM)—a potentially life-threatening condition during pregnancy, childbirth or after termination of pregnancy—can be used as a quality indicator of the health care provided to mothers and children. The aim of this study was to investigate the SMM rate and the main factors associated with this condition among women living in the Federal District, Brazil.

**Methods:**

We conducted a cross-sectional population-based sample survey using a structured questionnaire about the sociodemographic characteristics of the participants’ families. The data investigated included receipt of financial aid from the Federal Government, age, race, maternal educational level, prenatal care, mode of delivery, and serious complications during pregnancy and postpartum (SMM). 1042 mothers of children up to 1 year old were interviewed, representing a weighted estimated population of 36,724 mothers. The sample was representative of the whole Federal District state.

**Results:**

Mothers were between 19 and 34 years old (69%), most of them were brown or black (59.7%), and they had more than 9 years of education (81.2%). Prenatal care was adequate for 91.9% of them, the most common mode of delivery was Cesarean section (61.3%), and most deliveries took place in public hospitals (57.3%). The prevalence of low birth weight (< 2,500 g) was 8.1%. We found 2072 events of SMM in 2060 mothers (SMM rate: 5.6%). There was an association between higher occurrence of SMM and older age (OR: 1.40; 1.26–1.56), lower maternal educational level (OR: 3.29; 2.78–3.90), and inadequate prenatal care (OR: 1.28; 1.09–1.51). Receipt of financial aid was also associated to increased risk for SMM (OR: 1.31; 1.16–1.48). Cesarean section and low birth weight reduced the risk of SMM (decrease of 49.0% and 46.0%, respectively).

**Conclusions:**

The SMM rate in the Federal District was positively associated with higher maternal age, lower maternal educational level, inadequate prenatal care, and government financial aid program. Conversely, SMM was inversely associated with Cesarean delivery and low birth weight. This study showed that specific demographic groups are at higher risk for SMM. Therefore, actions should be focused primarily on those groups for greater effectiveness at reducing maternal mortality and providing better quality of maternal health care.

## Introduction

The World Health Organization (WHO) defines maternal death as "the death of a woman during pregnancy or within a period of 42 days after the end of the pregnancy, regardless of the pregnancy duration or location, from any cause related to or aggravated by pregnancy or by measures related to it, but not from accidental or incidental causes"[1]. Approximately 1,000 women die from pregnancy-related causes every day, and 10 million women have complications related to pregnancy worldwide every year[2]. Furthermore, 99% of maternal deaths occur in low income countries[3]. In Brazil, where almost all deliveries occur in hospitals (98.08%), the maternal mortality rate is estimated at 68.2 deaths per 100,000 live births[4].

Women who survived the death risk conditions arising from pregnancy and childbirth complications have many aspects in common with women who died from these complications. Such similarities led to the development of the concept of severe morbidity in maternal health[5].

Servere maternal morbidity is a potentially life-threatening condition during pregnancy, childbirth or after termination of pregnancy from which maternal near miss cases would emerge[6]. It generally describes a condition that did not result in damage or disease, but had the potential to do so, including its most severe group, the maternal near miss[7]. It is estimated that for every maternal death there are, on average, 15 cases of near miss worldwide[8]. In Brazil, the maternal near-miss-to-mortality ratio is 3.08%[9].

The most common causes of SMM are severe postpartum hemorrhage, severe pre-eclampsia, sepsis, uterine rupture, and critical interventions (blood transfusions, laparotomy and admission to the intensive care unit). These factors are often associated with prenatal care quality and women care during childbirth and the perinatal period[6].

The study of the similarities, differences, and the relationship between the women who died and those with life threatening complications who survived provides a more complete evaluation of the quality of maternal health care. This investigation enables the identification of corrective actions to reduce mortality and long-term morbidity[5,7].

Accordingly, SMM can be used as a quality indicator of the mother and child health care, thus becoming a very important tool to reduce maternal mortality and complications during the pregnancy-postpartum period[10]. Therefore, the aim of this study was to investigate the SMM rate and the main factors associated with it among women living in the Federal District, Brazil.

## Methods

We conducted a cross-sectional population-based study using data from the survey "Neonatal Call: diagnosis of health conditions and health care of maternal and child population in the Federal District (FD)". Data were collected in August 2011, during the "D-Day" of the polio vaccination campaign in the Federal District, with mothers of children under 1 year of age, covering a large portion of the target population (99.4%)[11].

Cluster sampling was performed using two-stage proportional probability sampling. For sample size calculation, we considered a conservative prevalence of 50%, considering that several indicators were investigated, a confidence level of 95%, and a maximum sampling error of 4%. The sample calculation resulted in a sample size of 1,170 mother-child pairs after adjusting for a sampling loss of 30% and correcting the effect of sample design to 1.5.

The first stage consisted of the selection of 26 vaccination centers located in the FD. The second stage consisted of the systematic selection of mother-child pairs in the vaccination centers on the date of the survey. The pairs were selected at predetermined intervals throughout the day to achieve the target sample at each vaccination center and to ensure equal probability of participation, regardless of the time of attendance to the vaccination center. When attendance was lower than expected, the selection interval was reduced to achieve the target sample, preserving the inclusion criteria and sample randomness. The eligibility criteria were mother-child pairs whose children were under one year old and were accompanied by their mothers; absence of condition or immobilization that could interfere with weight and height measurements; not being a twin or adopted child. If the eligible mother had more than one child under one year old, the oldest one was selected. After sample losses, 1,042 mother-child pairs were investigated, which did not affect the sample representativeness according to sample loss criteria. Those 1,042 mother-child pairs represented a total of 36,724 pairs in the general population of the Federal District.

Based on the sample design, each case represented a parcel of the region’s population. This was taken into account in the results, weighting the values in the statistical analysis based on the representativeness of each subject to the total population.

The weighting factor was calculated as the number of children under 1 year old vaccinated in the second phase of the poliomyelitis vaccination campaign in the FD, using the data provided by the Federal District Health Department, in addition to the sample design. This campaign’s vaccination coverage was 98% among children under 12 months in the FD[12]. Such high coverage helps reduce selection biases in this type of data collection strategy[13]. For data collection, we used a previously tested structured questionnaire based on the survey used in the “Neonatal Call: Legal Amazon/Northeast” conducted by the Brazilian Ministry of Health in 2010[14]. The questionnaire consisted of closed questions about family sociodemographic characteristics, receipt of financial aid from the Federal Government (*Bolsa Familia* Program), age, race, and maternal educational level, prenatal care, mode of delivery, and serious complications during pregnancy and postpartum (SMM).

The following criteria were used for the classification of SMM, based on Say et al.[7] and Souza et al.[15]: eclampsia, hysterectomy (due to pregnancy complications), blood transfusion, and transfer to the intensive care unit (ICU) up to 42 days after delivery[6]. Clinical and laboratory criteria were not used because these data were not collected due to limitations of the study design, which did not include laboratory tests or access to maternal medical records. The WHO criterion for blood transfusion is more than five units of transfused blood. However, this study considered the positive report of blood transfusion as the event occurrence regardless of the number of transfused bags because the patient was not aware of this information. Such adaptation does not restrict the measurement of severe morbidity events[16,17].

Prenatal care was considered adequate when pregnant women attended six or more medical visits during pregnancy[18] regardless of the period of attendance.

The SPSS 22.0 software was used to perform the bivariate analysis (Chi-square test with Pearson's correction) and logistic regression. The significance level was set at p <0.05. The logistic regression variables were considered eligible for the bivariate analysis when their p-value was <0.2. This study was approved by the Research Ethics Committee of the University of Brasília under the protocol no. 130/10, February 9th, 2011. All participants signed an informed consent form.

## Results

Most mothers were between 19 and 34 years old (69%), had brown or black skin color (59.7%), and had more than 9 years of education (81.2%). Prenatal care was considered adequate (six or more visits) for 91.9% of women, the most common mode of delivery was Cesarean section (61.3%), and the most deliveries occurred in public hospitals (57.3%). The prevalence of low birth weight (< 2.500 g) was 8.1%. We found 2072 events of SMM in 2060 mothers (non-weighted population: 46 events in 42 mothers/survey cases), with a total SMM rate (SMMR) of 5.6% (Table 1).

**Table 1:** Characteristics of mothers and children, total population and severe maternal morbidity cases; Weighted percentages, Federal District, 2011.

	Total population			Severe maternal morbidity cases					
	*n*	%	CI (95%)	*n*	%	OR*	CI 95%	OR adj*	CI 95%
**Age (years)**									
≤ 18	107	9.0	8.7–9.3	8	3.3	0.58	0.48–0.71	0.42	0.34–0.52
19–34	755	69.0	68.5–69.5	29	5.6	1		1	
≥ 35	180	22.0	21.5–22.4	9	6.9	1.25	1.13–1.38	1.40	1.26–1.56
**Color**									
White	304	35.4	34.9–35.9	13	5.0	1		1	
Brown/black	688	59.7	59.2–60.2	28	5.9	1.18	1.07–1.30	1.05^§^	0.94–1.18
Other	50	4.9	4.7–5.1	5	7.4	1.51	1.25–1.84	1.40	1.15–1.71
**Education (years of study)**									
≤ 8	229	18.8	18.4–19.2	13	7.8	1.88	1.67–2.12	3.29	2.78–3.90
9–11	512	43.4	42.9–43.9	20	5.9	1.38	1.25–1.54	2.14	1.87–2.45
≥ 12	301	37.8	37.3–38.3	13	4.3	1		1	
**Adequate prenatal care**									
Yes	936	91.9	91.6–92.8	40	5.6	1		1	
No	106	8.1	7.8–8.4	6	6.5	1.18	1.01–1.38	1.28	1.09–1.51
**Mode of Delivery** ^1^									
Vaginal	451	38.7	38.2–39.2	20	7.0	1		1	
Cesarean	582	61.3	60.8–61.8	26	4.8	0.67	0.61–0.73	0.51	0.46–0.57
**Delivery place** ^2^									
Public hospital	680	57.3	56.8–57.8	30	5.5	1			
Private hospital	350	42.7	42.2–43.2	16	5.9	1.07^$^	0.98–1.17		
**Child birth weight (g)** ^3^									
< 2,500	78	8.1	7.8–8.4	3	3.4	0.57	0.46–0.70	0.54	0.44–0.66
≥ 2,500	946	91.9	91.6–92.2	43	5.9	1		1	
**Financial aid**									
Received	180	18.0	17.6–18.4	8	7.5	1.48	1.33–1.64	1.31	1.16–1.48
Did not receive/did not know	862	82.0	81.6–82.4	38	5.2	1		1	
**Total**	1042	100		46	100				
**Severe maternal morbidity**									
Eclampsia	17	2.3	2.15–2.45						
Blood transfusion	13	1.1	0.99–1.21						
Hysterectomy	16	2.2	2.05–2.35						
Transfer to ICU	2	0.1	0.08–0.14						
**Severe maternal morbidity cases**	46	5.6	5.36–5.84						

^**1**^- *n* = 1033;

^**2**^- *n* = 1030;

^**3**^- *n* = 1024

*- all *p* < 0.05, except when indicated otherwise

^**$**^- *p* = 0.12

^**§**^- *p* = 0.34

The odds ratio (OR) showed that non-white women with lower educational level, aged 35 years old and older, who did not receive adequate prenatal care, and received financial aid from the government had a higher risk for SMM. Undergoing a Caesarean section, being an adolescent mother, and having a child with low birth weight were protective factors. There was no statistical difference in terms of location of delivery (public or private hospital; p = 0.12) and SMM.

The ORs adjusted by logistic regression showed the same trends of the gross analysis, except for black skin color, which lost significance in the adjusted analysis. There was an association between higher occurrence of SMM and older age (OR: 1.40), lower maternal education (OR: 3.29), and inadequate prenatal care (OR: 1.28). Financial aid from the government also resulted in increased risk for SMM (OR: 1.31). Undergoing a Cesarean section and having a low-birth-weight child reduced the risk for SMM (49% and 46% risk reduction, respectively).

## Discussion

Studies investigating SMM and maternal near miss (MNM)—which is the most extreme group within the severe morbidity spectrum—have been mainly conducted in low income countries[19,20] because most maternal deaths occur in these countries. The objective of these studies was to identify cases and support programs to reduce maternal morbidity and mortality.

Brazil was one of the pioneers in such studies, participating in research to validate the criteria currently used by the WHO to define SMM and MNM[7]. Several Brazilian studies have been published demonstrating the feasibility and effectiveness of this method[21,22,23]. However, these studies are limited because they were conducted in few health care facilities without a national scale and showing a great variation of values. In addition, the strict concept of MNM was developed in a hospital setting, at secondary and tertiary levels. Therefore, this concept is still being adjusted to measure data from more limited settings[24].

Maternal mortality is intrinsically related to socioeconomic conditions. When we analyze severe morbidity rates in different world regions, we find the same pattern, in which low income countries displays higher rates than high income countries, reflecting the availability and access to health services and pointing out that, along with local strategies, improving techniques and personnel qualification, the lowering of maternal mortality and morbidity is dependent on the improvement of the socioeconomic situation.

According to a systematic review[17], near miss rates by region denote this association. While in Africa the value ranged between 0.05–14.98% and in Latin America and the Caribbean between 0.34%– 4.93%, in high income regions the range was always lower: Europe, 0.04–0.79%; North America, 0.07–1.38%; Australia, 1.25%. In another study, De Mucio et al.[25] found out, in 11 Latin American maternity hospitals, a MNM prevalence of 1.29%.

Worlwide, many countries encourage pregnant women to deliver in health facilities. While this policy might reduce delays in the identification and management of peripartum complications, it might also lead to the service overload, which, in most cases, is already insuficiente[17].

It is important to note that, as showed in a systematic review by Tunçalp et al.[17], studies were mainly retrospective crosssectional, using data mainly from tertiary-care hospitals. Although it provides a better data collection, the results might be different from a community setting, since the population selection is biased, impairing the external validity for the total population.

However, several countries have national health information systems that can be used for comparison of major regions or cities, broadening the indicator to a more community related point-of-view. Using this approach, Sousa et al.[26], using the Hospital Information System (SIH) to gather SMM information in all Brazilian capitals, found a SMMR of 5.5% (5.29–5.71) in the FD, which is close to our study result (5.6%; 5.36–5.84), but higher than the national mean (4.4%; 4.38–4.48).

Amaral et al.[27] reported a SMMR of 3.54% in Campinas (SP) and Moraes et al.[22] found an incidence of 1.43% in a longitudinal study conducted in São Luís (MA), Brazil. The difference in these values may have been caused by the characteristics of the health care facilities where the studies were performed (that is, primary health care facilities or reference maternities). Silva et al.[9] stated that MNM and SMM also reflects the availability of services and access to health care in more complex hospitals. Therefore, its use as an indicator would be more appropriate to evaluate the quality of health care in comparison with higher income regions or countries.

In 2010, the Brazilian Ministry of Health conducted a broad research on prenatal care, childbirth, and under 1-year-old children in the Amazon and in the Northeast region[14]. One of the items evaluated was the occurrence of SMM, showing a total SMMR of 2.9%. Most states in these historically poorer regions had lower rates than that observed in our survey (4.8%). However, maternal mortality is lower in the FD than in these other locations, suggesting that, in the FD, these women might not have died—but suffered SMM because they received better health care and death was avoided. A study similar to ours conducted in a Brazilian state capital city (Natal-RN) found a SMMR of 4.1%, with the highest prevalence of severe morbidity observed in older, black/brown women with low socioeconomic status[28].

The nationwide study “Born in Brazil (*Nascer no Brasil*)” on maternal and children health during pregnancy and childbirth, investigating the more severe form of morbidity, maternal near miss cases, found a rate of 1,02%, which is higher in state capital cities (the FD is the capital of Brazil and it has the same characteristics of other Brazilian state capital cities)[9]. The same study identified a higher incidence of MNM in public hospitals and among mothers who have had Caesarean sections[9]. The MNMR was higher in the capital cities and reference centers (all of them are public hospitals in Brazil) because the population spontaneously seeks or is referred to such facilities when there have pregnancy complications.

Alongside our study, Domingues et al.[29] showed an association between the absence of antenatal care and SMM (OR 2.90; 0.94–8.92). It is noteworthy that the absence of antenatal care was associated with less years of schooling, with black or mixed skin colour, with not living with a partner and with having previous births, in accordance to our findings. On the other hand, eletive cesarean was a risk factor, whereas we found out the inverse association.

Our study showed no difference in the SMMR by hospital type, but Cesarean sections were a protective factor. Although cesarean delivery traditionally is a risk factor for SMM[22], mainly because of the surgical risk inherent to the procedure, a possible reason why a Cesarean section is a protective factor is because when it is an elective surgery, indicated according to medical criteria, this surgery protects mothers from eclampsia or other serious complications[30].

It is noteworthy the high Cesarean rate found in our study (61.3%). In Brazil, surgical delivery is highly valued among health professionals and, arguably, among women[7]. Most of these deliveries are booked in advance and both, the newborn and the mother, stay a few days longer in the hospital. During the hospital stay, several conditions are investigated and might be properly treated on time, preventing further complications and more severe outcomes. Although these characteristics, inherent to Brazil, may help to explain the Cesarean delivery and low birth weight—related to surgical delivery and the higher occurrence of preterm babies in Brazil—as protective factors, this subject needs to be further investigated.

A Cesarean section may increase in five-fold the chance of a woman to become a case of severe morbidity; however, this association may be influenced by confounding factors. Nevertheless, it is arguable whether the Caesarean is a risk factor for severe maternal morbidity, or if it is actually a consequence of this condition[22,24].

In our study, pregnancy and childbirth among teenagers was a protective factor, despite they had the highest percentages of inadequate prenatal care (27.2%) compared to the total sample (8.1%). It was also observed that there was increased risk of SMM (1.40) among mothers aged 35 years old or older. Analyzing the risk of serious maternal outcomes in extreme reproductive ages, Oliveira et al.[31] concluded that older age is an independent risk factor (OR 1.25; 1.07–1.45) for severe outcomes (SMM and death). Similar results were found by Dias et al.[32] (RR 1.6; 1.1–2.5) and in our study.

The variable with the highest risk related to SMM was maternal education, with gradual risk increase for the less educated groups. Among mothers who had eight years or less of education, the OR was more than three times higher. This was the most vulnerable group to the occurrence of SMM. The risk of SMM was also higher among mothers who received financial aid from the government. In fact, among women, low educational level and income are associated with worse health care and lower access to health services and information, which are related to a higher incidence of SMM[28,33]. The percentage of low birth weight children in the FD was 8.1%, which is similar to the national rate for the same year (9.9%)[4].

We found a high percentage of Cesarean sections (61.3%), which is higher than the national means reported by the DHS 2006 (43.8%)[34] and the Born in Brazil study (52.0%)[35] and much higher than the recommended by the WHO (10–15%)[36]. Brazil is experiencing an epidemic of surgical deliveries, and only 5% of women give birth without any medical intervention, such as episiotomy, Kristeller maneuver, use of forceps, among others[35]. In Brazil, Cesarean sections are higher among white mothers with higher educational level, higher socioeconomic status, and those who give birth in private hospitals[34]. The FD is the federation unity with the highest income per capita, human development index, and educational level in the country[4], in addition to broad private health insurance coverage[37]: all these factors are related to Cesarean delivery[35]. The high prevalence of Cesarean sections in the FD and its associations need to be further investigated to indicate paths to reduce these numbers.

Soma-Pillay & Pattinson[38] showed that in 83% of the severe morbidity (near miss) cases occurred at least one factor delaying access to adequate care, such as lack of knowledge of the problem, inadequate antenatal care, delay in patient admission, referral and treatment. The leading causes of near miss, as a consequence of this delay were obstetric haemorrhage, hypertension/pre-eclampsia, and medical and surgical conditions. Thus, educating the community about symptoms and complications related to pregnancy might prevent maternal morbimortality, as well as additional visits in the third trimester.

In agrement with Domingues et al.[29], our findings showed that severe maternal morbidity risk factors—in a context of universal health care (Brazilian National Health System) and specially antenatal care—are associated with barriers to health services access, indicating social, economic and demographics vulnerabilities.

## Conclusion

It is noteworthy that this is a cross-sectional study with limitations to causal inference. Also, the main concern in our research was that it was based on information provided by the mothers, without access to medical records to confirm diagnoses and procedures, potentially leading to memory bias. Although we did take that into account, one of the study purposes was to use an easy and pragmatic approach to data collection that might be reproducible in any setting, regardless of technical and economic conditions.

SMM is a new indicator whose applicability has been expanding worldwide as a quality indicator of maternal health care. Its use brings advantages, such as easy recruitment of a large number of subjects, thus allowing the identification of corrective actions to be taken to reduce mortality and long-term morbidity.

SMM in the FD was positively associated with higher maternal age, lower maternal educational level, inadequate prenatal care, and financial aid program. SMM was inversely associated with Cesarean delivery and low birth weight. This study also indicates the high prevalence of Cesarean sections in the FD, a fact that needs to be further investigated.

SMM needs to be understood not only as a biological or a health care phenomenon, but also as a socioeconomic phenomenon, where the worst conditions increase the risk of severe pregnancy complications, thus suggesting that the actions to fight maternal mortality should focus primarily on specific demographic groups for greater effectiveness.

Importantly, data on SMM are scarce and necessary for comparisons and decision-making in central levels aimed at reducing maternal mortality and achieving better quality of maternal health care.

## Supporting information

S1 Table Responses to the "Neonatal Call: diagnosis of health conditions and health care of maternal and child population in the Federal District” survey. (SAV)
